# Intakes of folate, vitamin B6, and vitamin B12 and cardiovascular disease risk: a national population-based cross-sectional study

**DOI:** 10.3389/fcvm.2023.1237103

**Published:** 2023-11-14

**Authors:** Jiamin Huang, Pipasha Khatun, Yuqing Xiong, Bingrui Liu, Yisu Zhao, Quanjun Lyu

**Affiliations:** ^1^ Department of Nutrition and Food Hygiene, College of Public Health, Zhengzhou University, Zhengzhou, China; ^2^ Department of Clinical Nutrition, The First Affiliated Hospital of Henan Polytechnic University, Jiaozuo, China; ^3^ Department of Public Health, Zhengzhou Shuqing Medical College, Zhengzhou, China

**Keywords:** cardiovascular disease, diet, folate, vitamin B6, vitamin B12

## Abstract

**Background:**

Only a few studies that investigated dietary intakes of folate, vitamin B6, and vitamin B12 in relation to cariovascular disease (CVD). This study aimed to assess the association of dietary folate, vitamin B6, and vitamin B12 with CVD in the United States population.

**Methods:**

A cross-sectional analysis of 65,322 adults aged ≥ 20 years who participated in the Third National Health and Nutrition Examination Survey (NHANES III) and NHANES 1999–2018. Before 2003, dietary intake data were assessed using a 24-hour dietary call, and two 24-hour dietary calls were used during 2003 and 2018. Odds ratios and 95% confidence intervals (CIs) for CVD associated with dietary folate, vitamin B6, and vitamin B12 were estimated using multivariate logistic regression models.

**Results:**

Dietary vitamin B6 intake were inversely associated with the odds of CVD. In males, the multivariable OR for the highest vs. lowest quartiles of vitamin B6 was 0.77 (95%*CI*: 0.61–0.97, *P*_trend _= 0.013) for the odds of CVD. In females, the adjusted OR for the highest quartile of vitamin B6 compared with the lowest quartile was 0.73 (95%CI: 0.56–0.95, *P*_trend _= 0.038) for the odds of CVD. No significant association was observed between dietary folate and vitamin B12 intakes and the odds of CVD.

**Conclusions:**

Our findings indicate that higher intake of dietary vitamin B6 may be associated with lower prevalence of CVD, suggesting that dietary vitamin B6 has major public health implications in the prevention of CVD in the United States population.

## Introduction

Cardiovascular disease (CVD) exists as the leading cause of death globally, especially among adults in most of high-income countries, including the United States and Switzerland (1, 2). In recent decades, the prevalence of CVD has remained high, with approximately 17 million of CVD mortality each year globally (3). Specifically, men have much higher incidence rate of CVD than women (4). In order to reduce morbidity and mortality, early detection and intervention of risk factors is critical (5). Numerous studies have demonstrated the value of changing poor dietary habits and certain nutrient intake to prevent CVD (6–8).

Certain B vitamins play vital roles in the degradation of blood homocysteine, such as folate, vitamin B6 and vitamin B12 (9, 10), and the deficiency of which is associated with the development of CVD (11–13). Multiple epidemiological studies have reported hyperhomocysteinemia as an independent risk factor for cardiovascular disease (14, 15). Several studies have found that supplementation with B vitamins (folate, vitamin B6, and vitamin B12), in addition to normal dietary intake, can reduce plasma homocysteine levels and the prevalence of hyperhomocysteinemia, which may be beneficial in reducing CVD risk (16–18). However, this hypothesis is still controversial, and a number of randomized controlled trials and meta-analyses have not confirmed that B vitamins treatment can reduce hyperhomocysteemia to provide cardiovascular protection (19, 20). This may be explained by different study populations, sample sizes and CVD outcomes in different studies.

Early meta-analyses suggested lowering plasma homocysteine could reduce the risk of CVD (21, 22). However, many epidemiologic studies have reported inconsistent findings regarding the relationship between dietary intake of B vitamins and CVD. For instance, the Japan Collaborative Cohort Study (23) found that higher dietary folate and vitamin B6 intakes was associated with a reduced risk of some CVDs, such as coronary heart disease, stroke, and heart failure. Whereas, the relationship between vitamin B12 and CVD was not observed. Bazzano et al. (24) reported an inverse relationship between dietary intake of folate and risk of CVD in the United States. Jeon et al. (25) observed that increased dietary vitamin B6 intake reduced the risk of CVD in Korean men through a prospective study. Conversely, in a Finnish study fund that vitamin B6 and vitamin B12 intakes was not associated with CVD risk in the elderly population (26). From the above, most of these studies on specific populations. Evidence among general adults is scares. Furthermore, although studies based on similar data have found that folate, vitamin B6 or vitamin B12 have an effect on death and high blood pressure, their effect on cardiovascular disease is unclear (27–29). Therefore, we investigated the association of dietary folate, vitamin B6, and vitamin B12 with the prevalence of CVD in a cross-sectional study of the general United States adult population using data from the National Health and Nutrition Examination Survey (NHANES).

## Methods

### Study population

The NHANES is a nationally representative sample of individuals surveyed by the National Center for Health Statistics (NCHS) of the Centers for Disease Control and Prevention (CDC). Ascertainment and data collection have been previously described (30) (last accessed 08-03-2022). Briefly, NHANES data were not obtained using a simple random sample. Rather, a complex, multistage, stratified probability sampling design was used to select a sample representative of the civilian noninstitutionalized household population of the United States. It is designed to assess the health and nutritional status of civilians in the noninstitutionalized United States. Written informed consent for the survey were obtained from all participants, and study protocols was approved by the institutional review board of the NCHS.

Subjects for this study were participants in the NHANES Ⅲ (1988–1994) and ten cycles from 1999 to 2018 epidemiologic follow-up studies of the NHANES. Participants aged ≥ 20 years were included in the analysis. The study originally included 132,627 United States residents who attended medical examination at NHANES from 1988 to 2018. Of these, some participants who were younger than 20 years or who lacked data on dietary folate, vitamin B6, or vitamin B12 intakes were excluded, yielding a final sample size of 65,322 adults.

### Assessment of dietary folate, vitamin B6, and vitamin B12 intake

Data on dietary folate, vitamin B6, and vitamin B12 intakes from foods were evaluated for each participant by trained interviewers through 24-hour dietary recall. During NHANES Ⅲ and 1999–2002 surveys, each person was asked to complete a 24-hour dietary recall during the Mobile Examination Center (MEC) visit. From 2003 to 2018, two dietary recalls were conducted. The first collection was conducted in the MEC and the second was collected by telephone 3–10 days later. Dietary intakes of folate, vitamin B6, and vitamin B12 were assessed using the United States Department of Agriculture (USDA) Food and Nutrient Database for Dietary Studies (FNDDS), versions 1.0–5.0 (31).

### Outcome aschertainment

CVD is a class of diseases involving the heart and blood vessels, defined as suffering from any of the following diseases, including coronary heart disease (CHD), congestive heart failure (CHF), angina pectoris, myocardial infarction and stroke. During the interview, information on CVD was obtained through self-reported physical diagnoses.

### Covariates

The main characteristics data of participants were collected, including age, sex (men or women), race/ethnicity (Hispanic, non-Hispanic white, non-Hispanic black, and Race-including multi-racial), alcohol drinking (never, low-to-moderate, or heavy), smoking (nonsmokers, former smokers, or current smokers), family income and physical activity. The ratio of family income to poverty was calculated by dividing household income by the poverty guidelines for the year of the survey. The ratio of family income to poverty was categorized as less than or equal to 1, 1 to 3, or greater than 3. Leisure-time physical activity was defined as the product of metabolic equivalent value [MET]. Standardized questionnaires were used to collect self-reported physician diagnosed CVD (yes or no), type two diabetes (yes or no) and hypertension (yes or no). The computational formula of BMI is weight (kilograms) divided by height (meters squared).

### Statistical analysis

Categorical variables were presented as numbers (percentages), and continuous variables were expressed as means (standard deviations). Missing values are listed in Table 1.

**Table 1:** Main characteristics of the study population.

	Total sample	Non-CVD	CVD
	*N* = 65,322	*N* = 58,489	*N* = 6,833
Age, year	49.18 (18.50)	47.09 (17.87)	67.16 (13.22)
Sex			
Female	34,039 (52.11%)	31,056 (53.10%)	2,983 (43.66%)
Male	31,283 (47.89%)	27,433 (46.90%)	3,850 (56.34%)
Race			
Hispanic	17,000 (26.02%)	15,769 (26.96%)	1,231 (18.02%)
Non-hispanic white	28,652 (43.86%)	24,874 (42.53%)	3,778 (55.29%)
Non-hispanic black	14,846 (22.73%)	13,361 (22.84%)	1,485 (21.73%)
Other race—including multi-racial	4,824 (7.38%)	4,485 (7.67%)	339 (4.96%)
Drinking			
Never	9,914 (24.59%)	8,597 (23.58%)	1,317 (34.15%)
Low to moderate	8,618 (21.38%)	7,464 (20.47%)	1,154 (29.92%)
Heavy	21,781 (54.03%)	20,395 (55.94%)	1,386 (35.93%)
Missing	25,009	22,033	2,976
Smoking			
Never	14,457 (22.15%)	13,116 (22.44%)	1,341 (19.63%)
Former	16,257 (24.90%)	13,437 (22.99%)	2,820 (41.28%)
Current	34,569 (52.95%)	31,899 (54.57%)	2,670 (39.09%)
Missing	39	37	2
Ratio of family income to poverty			
≤1	12,543 (21.05%)	11,071 (20.74%)	1,472 (23.73%)
1–3	25,850 (43.38%)	22,723 (42.56%)	3,127 (50.41%)
>3	21,196 (35.57%)	19,592 (36.70%)	1,604 (25.86%)
Missing	5,733	5,103	630
Diabetes	7,268 (11.13%)	5,224 (8.93%)	2,044 (29.91%)
Hypertension	21,494 (32.90%)	16,764 (28.66%)	4,730 (69.22%)
BMI, kg/m^2^	28.25 (6.04)	28.14 (6.02)	29.25 (6.11)
Leisure-time physical activity, MET	16.79 (13.07)	16.91 (13.12)	15.40 (12.41)

Odds ratios and 95% CIs for CVD associated with dietary folate, vitamin B6, and vitamin B12 were estimated using multivariate logistic regression models. We divided dietary nutrients (i.e., dietary folate, vitamin B6, and vitamin B12) into quartiles and selected the first quartile as the reference group. Two models were gradually introduced: Model 1 was an unadjusted coarse model. Model 2 was adjusted for certain potential confounders, including age, race/ethnicity, BMI, family income-poverty ratio, smoking status, drinking, leisure-time physical activity, total energy intake, and comorbidities.

We used stratified analyses to assess the potential effect modification by age (<65 or ≥65 years), current smoker (yes or no), current drinker (yes or no), and BMI (<30 kg/m^2^ or ≥30 kg/m^2^). Each potential modifier was examined separately by including a multiplicative interaction term (i.e., continuous dietary nutrients intake parameters* potential modifier) in the multivariate model.

The R 4.0.2. (R Core Team, Vienna, Austria) was used to perform all statistical analyses. A two-sided test was used for this analysis, and *P* values < 0.05 were considered statistically significant.

## Results

### Participant characteristics

As shown in Table 1. A total of 65,322 individuals were included in this study, with an average age of 49.18 (SD: 18.50) years. A total of 6,833 CVD cases were identified, including 2,983 in female and 3,850 in men. Compared with participants with non-CVD, those with CVD were older, had a greater proportion of men and non-Hispanic white people, were more likely to be non-current smokers, were more likely to have diabetes and hypertension, and had lower family incomes. Additionally, participants with CVD are more likely to be obese and have low physical activity.

### Dietary folate, vitamin B6, and vitamin B12 with CVD

The sex-related associations between dietary folate, vitamin B6, and vitamin B12 intakes and CVD are shown in Table 2. Among males, an inverse association was observed between vitamin B6 intake and the prevalence of CVD, with an adjusted OR for the highest quartile vs. the lowest quartile was 0.77 (95%CI: 0.61–0.97, *P*_trend _= 0.013). Before adjustment for potential confounders, we found an association between dietary folate and vitamin B12 intakes and CVD, but after adjustment, no significant relationships were observed between dietary folate and vitamin B12 intakes and CVD. The multivariable OR for the highest vs. lowest quartiles in men were 1.09 (95%CI: 0.85–1.42, *P*_trend _= 0.774), and 0.91 (95%CI: 0.74–1.13, *P*_trend _= 0.418) for dietary folate and vitamin B12, respectively. Among females, we observed similar results. For dietary folate, vitamin B6, and vitamin B12, adjusted ORs for the highest quartile compared with the lowest quartile in women were 0.76 (95%CI: 0.56–1.01, *P*_trend _= 0.111); 0.73 (95%CI: 0.56- 0.95, *P*_trend _= 0.038); 0.79 (95% CI: 0.62–1.00, *P*_trend _= 0.073).

**Table 2:** Odds ratio (ORs) and 95% CIs for CVD according to quartiles of dietary B vitamins.

	Quartile of nutrient intake				*P*_trend_
	Q1	Q2	Q3	Q4	
Men					
Dietary folate					
Crude model (*n* = 31,264)	Ref	1.15 (1.03–1.28)	1.04 (0.93–1.16)	0.83 (0.74–0.93)	<0.001
Multivariable model (*n* = 16,683)	Ref	1.11 (0.90–1.37)	1.03 (0.82–1.29)	1.09 (0.85–1.42)	0.774
Vitamin B6					
Crude model (*n* = 31,275)	Ref	0.89 (0.80–0.99)	0.87 (0.78–0.97)	0.66 (0.59–0.74)	<0.001
Multivariable model (*n* = 16,683)	Ref	0.90 (0.74–1.08)	0.77 (0.63–0.94)	0.77 (0.61–0.97)	0.013
Vitamin B12					
Crude model (*n* = 30,909)	Ref	0.94 (0.85–1.05)	0.95 (0.85–1.06)	0.79 (0.70–0.88)	<0.001
Multivariable model (*n* = 16,651)	Ref	0.96 (0.79–1.16)	0.95 (0.79–1.16)	0.91 (0.74–1.13)	0.418
Women					
Dietary folate					
Crude Model (*n* = 34,026)	Ref	1.20 (1.05–1.38)	1.13 (0.99–1.30)	0.84 (0.72–0.97)	0.013
Multivariable Model (*n* = 18,411)	Ref	0.84 (0.66–1.07)	0.85 (0.66–1.10)	0.76 (0.56–1.01)	0.111
Vitamin B6					
Crude model (*n* = 34,025)	Ref	0.94 (0.83–1.08)	0.92 (0.81–1.05)	0.74 (0.65–0.86)	<0.001
Multivariable Model (*n* = 18,413)	Ref	0.79 (0.63–0.99)	0.78 (0.62–0.99)	0.73 (0.56–0.95)	0.038
Vitamin B12					
Crude model (*n* = 33,656)	Ref	0.88 (0.77–1.01)	0.93 (0.81–1.06)	0.82 (0.72–0.94)	0.015
Multivariable model (*n* = 18,364)	Ref	0.84 (0.67–1.05)	0.84 (0.67–1.05)	0.79(0.62–1.00)	0.073

Crude OR: did not adjust anything.

Multivariable OR: adjusted for age, race/ethnicity, BMI, family income-poverty ratio, smoking status, drinking, leisure-time physical activity, total energy intake, hypertension, and diabetes.

### Stratified analysis among male group

For male participants, stratified analysis by age indicated that no statistically significant difference between dietary folate and CVD. Whereas, there was an interaction between the two groups (*P* for interaction < 0.001). Stratified by smoking status, drinking or not, and BMI, the results showed that dietary folate intake was not significantly associated with CVD (all *P*-values > 0.05) (Supplementary Table S1).

As shown in Table 3, when stratified based on age, higher dietary vitamin B6 intake was associated with a lower prevalence of CVD among male participants younger than 65 years, with the adjusted OR for the highest quartile vs. the lowest was 0.60 (95%CI: 0.42–0.86, *P*_trend _= 0.002); no statistically significant results were found in men with a weight ≥ 65 years. However, there was a significant interaction between dietary vitamin B6 intake and age (*P* for interaction < 0.001). Stratified by smoking status, increased dietary vitamin B6 intake was found to be associated with a decreased odds of CVD among current smokers, with an adjusted OR of 0.66 (95%CI: 0.44–0.99, *P*_trend_ = 0.017); According to alcohol consumption stratified, we found that vitamin B6 intake was inversely associated with CVD in drinking men, with an adjusted OR of 0.73 (95%CI: 0.57–0.94, *P_t_*_rend_ = 0.007), comparing the highest quartile with the lowest; We found no statistically significant results in analyses stratified by BMI. There was no relationship between dietary vitamin B12 and CVD in men by stratified analysis (Supplementary Table S2).

**Table 3:** Stratified analyses of the associations between dietary vitamin B6 intake with CVD in men.

	Quartile of dietary vitamin B6 intake				*P*_trend_	*P*_interaction_
	Q1	Q2	Q3	Q4		
Age						
<65 y	Ref	0.80 (0.60–1.06)	0.58 (0.43–0.79)	0.60 (0.42–0.86)	0.002	<0.001
≥65 y	Ref	1.11 (0.85–1.45)	0.87 (0.66–1.16)	0.89 (0.66–1.22)	0.195	
Current smoker						
Yes	Ref	0.90 (0.65–1.24)	0.68 (0.48–0.96)	0.66 (0.44–0.99)	0.017	0.195
No	Ref	0.96 (0.75–1.21)	0.81 (0.63–1.04)	0.81 (0.61–1.08)	0.080	
Current drinker						
Yes	Ref	0.87 (0.71–1.05)	0.75 (0.60–0.92)	0.73 (0.57–0.94)	0.007	0.878
No	Ref	1.04 (0.70–1.55)	0.86 (0.55–1.35)	1.02 (0.61–1.71)	0.818	
BMI						
<30 kg/m^2^	Ref	0.98 (0.76–1.26)	0.82 (0.64–1.07)	0.81 (0.60–1.10)	0.093	0.464
≥30 kg/m^2^	Ref	0.79 (0.59–1.07)	0.79 (0.57–1.09)	0.78(0.54–1.12)	0.231	

Adjusted for age, race/ethnicity, BMI, family income-poverty ratio, smoking status (not for smoke stratified analysis), drinking (not for drinking stratified analysis), leisure-time physical activity, total energy intake, hypertension, and diabetes.

### Stratified analysis among female group

The results of stratified analysis of women are presented in Table 4; Supplementary Tables S3, S4. For female participants, the associations of dietary folate and vitamin B12 intakes with CVD were similar across subgroups. Linear trends in the reduction in the prevalence of CVD with increasing dietary folate and vitamin B12 intakes were observed only in never-smokers, and the linear trends were all statistically significant (all *P* for trend < 0.05). Stratified by age, we observed a marginally negative association between vitamin B6 intake and CVD in older adults (OR_Q4 vs. Q1_ = 0.70, 95%CI: 0.50–1.00, *P*_trend _= 0.045) (*P* for interaction = 0.364). The results of stratified analysis by smoking status showed that higher vitamin B6 intake was associated with decreased the odds of CVD among never-smoking women (OR_Q4 vs. Q1 _= 0.57, 95%CI: 0.39–0.85, *P*_trend_ = 0.004).

**Table 4:** Stratified analyses of the associations between dietary vitamin B6 intake with CVD in women.

	Quartile of dietary vitamin B6 intake				*P*_trend_	*P*_interaction_
	Q1	Q2	Q3	Q4		
Age						
<65 y	Ref	0.74 (0.52–1.04)	0.73 (0.52–1.04)	0.67 (0.44–1.00)	0.073	0.364
≥65 y	Ref	0.85 (0.63–1.16)	0.76 (0.55–1.04)	0.70 (0.50–1.00)	0.045	
Current smoker						
Yes	Ref	0.82 (0.60–1.12)	0.99 (0.72–1.36)	0.95 (0.66–1.36)	0.867	0.251
No	Ref	0.79 (0.56–1.10)	0.64 (0.46–0.91)	0.57 (0.39–0.85)	0.004	
Current drinker						
Yes	Ref	0.99 (0.75–1.31)	0.90 (0.66–1.23)	0.84 (0.59–1.20)	0.287	0.393
No	Ref	0.81 (0.59–1.11)	0.83 (0.60–1.16)	0.74 (0.50–1.07)	0.152	
BMI						
<30 kg/m^2^	Ref	0.87 (0.63–1.19)	0.76 (0.55–1.05)	0.70 (0.49–1.01)	0.049	0.941
≥30 kg/m^2^	Ref	0.72 (0.52–1.00)	0.82 (0.59–1.14)	0.78(0.53–1.13)	0.385	

Adjusted for age, race/ethnicity, BMI, family income-poverty ratio, smoking status (not for smoke stratified analysis), drinking (not for drinking stratified analysis), leisure-time physical activity, total energy intake, hypertension, and diabetes.

## Discussion

This large cross-sectional study focused on the association of dietary folate, vitamin B6, and vitamin B12 intake with CVD in a representative general adult population in the United States. The findings from this study indicate that intake of vitamin B6 was inversely associated with the prevalence of CVD, both in men and women. This negative relationship persisted after adjusting for various confounders. Nevertheless, no significant relationships were observed between dietary folate and vitamin B12 intake and CVD either in men or women.

Van Guelpen et al. (32) reported that dietary folate intake was not associated with acute myocardial infarction, which was similar to the results of this study. Dalmeijer et al. (33) investigated the relationship between dietary folate intake and CVD in 16,165 middle-aged and elderly Dutch women, and the findings were also similar to the present study, namely, that dietary folate was not associated with CVD risk in postmenopausal women. As well, a research on dietary intake of folate and the risk of acute myocardial infarction in elderly subjects reported the same results as this study (26). However, the results of our study are inconsistent with several previous studies on dietary folate intake and CVD risk. A large prospective cohort study of Finnish men found that high dietary folate intake was significantly inversely associated with a lower risk of CHD (34). A prospective cohort study of 40,803 subjects from the Japan Public Health Center showed that dietary intake of folate can reduce the risk of CHD (35). Similarly, an epidemiological study with data from NHANES I reported an inverse association between dietary intake of folate and CVD risk (24). The inconsistency of these findings may be due to the variation in study populations or research methods, or differences in dietary habits caused by geographical differences.

Currently, there is limited evidence on the relationship between dietary vitamin B6 and the risk of CVD. Our findings found that increased dietary vitamin B6 intake was associated with a lower prevalence of CVD the general United States population. In multivariate models, where we performed a stratified analysis, we observed a more significant negative association with CVD in drinking men and non-smoking women than in non-drinking men and smoking women. A dose-response meta-analysis including six prospective cohort studies reported a linear negative association between dietary vitamin B6 intake and the risk of CHD in the general population (36), similar to our results. Among them, Ishihara et al. (35) reported that dietary intake of vitamin B6 was inversely associated with CVD risk in middle-aged people using single vitamin supplements. A cohort study of middle-aged and elderly Chinese populations reported an inverse association between dietary vitamin B6 and cardiovascular disease (37). A prospective cohort study of 9,142 Koreans confirmed an inverse association between dietary vitamin B6 intake and the risk of CVD in men, however, this association was not observed in women, which is inconsistent with our findings (25). When analyses were stratified by age for the association between vitamin B6 and CVD, we found a significant interaction in men (*P*_interaction_ < 0.001) and no in women (*P*_interaction_ = 0.364). Considering the particularity of women's physiological status, we should pay attention to the effect of women's menopausal status on the results. Taken together, these studies are limited to specific populations. Interestingly, a recent large prospective cohort study assessed vitamin B6 status using plasma pyridoxal 5′ -phosphate (PLP), the active form of vitamin B6. A strong and independent inverse association of plasma PLP with the risk of cardiovascular events was found only in women (38). Potential explanations could be the difference in adjusted confounders and measured indicators.

In recent years, much attention has been paid to the potential mechanism by which vitamin B6 is associated with CVD. Two recent reviews describe advances in the cardioprotective role of vitamin B6 (39, 40). Vitamin B6 has been suggested to exert its cardiovascular protective effect possibly by inhibiting the inflammatory response through the kynurenine pathway, because in this pathway, PLP can convert kynurenine into various compounds that may have anti-inflammatory effects (41). Animal experiments have shown that increasing dietary vitamin B6 intake can significantly increase the levels of cardiac imidazole-dipeptides (including carnosine, anserine and homocarnosine) in rats or mice, which may play a protective role in heart through anti-inflammation, anti-oxidation, maintenance of physiological PH and so on (42–44). In addition, a cellular experimental study showed that vitamin B6 supplementation inhibited the nucleotide-binding oligomerization domain-like receptor family pyrin domain-containing 3 (NLRP3) inflammasome and reactive oxygen species production, which may indicate a novel mechanism of vitamin B6 antioxidant (45).

Our findings found no association between dietary vitamin B12 and the odds of CVD. To date, only a few literatures have reported the relationship between dietary vitamin B12 and CVD risk, and the reported results have been mixed. Voutilainen et al. (34) studied the relationship between dietary intake of vitamin B12 and CHD risk in Finnish men aged 42–60 years, and found that increased dietary intake was weakly associated with reduced risk of CHD. However, another study of older Finnish adults reported inconsistent results, observing that dietary vitamin B12 intake was not associated with a reduced risk of acute myocardial infarction (26). No clear relationship between dietary vitamin B12 intake and myocardial infarction has been observed in nested case-control studies based on the Swedish population (32). Interestingly, two prospective cohort studies, also from Japan, Cui (23) and Ishihara (35) reported conflicting results. Again, the heterogeneity of the study designs and study populations, making these studies impossible to directly compare with our study. In addition, it is well known that hypertension is a classic risk factor for CVD. We also examined the association between dietary B vitamins and hypertension in 55,569 adults from the NHANES and found that after adjustment, dietary intake of folate and vitamin B12 was inversely associated with hypertension (27). The conflicting results between the two studies may be due to differences in sample sizes and adjustment for confounding factors.

Our research has the following strengths. Foremost, this study used a representative United States non-institutionalized adult population as the research subject, and a stratified, multistage, complex probability sampling design was employed. Hence, our results are generally generalizable. In addition, a number of potential confounders, including demographic characteristics, smoking status, alcohol consumption and socioeconomic status, were adjusted when evaluating the association between dietary B vitamin intake and the prevalence of CVD. Further, the larger sample size and longer follow-up of NHANES allowed us to obtain more accurate estimates and conduct a series of analyses compared to other cross-sectional studies.

Our research also has several limitations. First, the dietary B vitamin intakes of each participant were obtained through one or two 24-hour dietary reviews without the use of a food-frequency questionnaire. However, as the dietary intakes of participants varied from day to day, daily dietary estimates may not reflect participants' long-term intake. However, measurement errors regarding dietary B vitamin intake due to estimates appear to be random, which tends to bias the resulting association in favor of null rather than spurious associations. Second, folate, vitamin B6, and vitamin B12 intakes were calculated using the USDA's FNDDS, which may have overestimated study participants' B vitamin intake because of changes in the content of B vitamins in foods, but this influence is integrity, may not lead to sharp changes in individual dietary intake rankings. Hence, we speculate that this limitation should not affect our findings. Finally, data on vitamin supplements were not collected in the NHANES study and were therefore not included in the analysis.

In conclusion, in the general US population, we observed an inverse association of dietary vitamin B6 with CVD. Our findings confirm that increasing dietary vitamin B6 intake reduces the risk of CVD, suggesting that dietary vitamin B6 has major public health implications in the prevention of CVD.

## Data Availability

The original contributions presented in the study are included in the article/**Supplementary Material**, further inquiries can be directed to the corresponding author.
