# Supplementary material for: Intakes of folate, vitamin B6, and vitamin B12 and cardiovascular disease risk: a national population-based cross-sectional study
Source: Front Cardiovasc Med. 2023 Nov 14;10:1237103. doi: 10.3389/fcvm.2023.1237103 (PMC10686214; doi:10.3389/fcvm.2023.1237103)
Supplement: Supplementary file 1 [file Table1.docx]

Supplementary Material

Intakes of Folate, Vitamin B6, and Vitamin B12 and Cardiovascular Disease Risk: A National Population-based Cross-sectional Study

Jiamin Huang^#^, Pipasha Khatun^#^, Quanjun Lyu^*^

^#^These authors contributed equally to this study.

*** Correspondence:** Quanjun Lyu, email: lqjnutr@zzu.edu.cn

**Supplemental Table 1. Stratified analyses of the associations between dietary folate intake with CVD in men**

|  | **Quartile of dietary folate intake** | | | | ***P_trend_*** | ***P_interaction_*** |
| --- | --- | --- | --- | --- | --- | --- |
|  | **Q1** | **Q2** | **Q3** | **Q4** |  |  |
| **Age** |  |  |  |  |  |  |
| <65y | Ref | 0.79(0.58-1.08) | 0.68(0.49-0.95) | 0.70(0.47-1.03) | 0.064 | <0.001 |
| ≥65y | Ref | 1.25(0.93-1.70) | 1.42(1.04-1.94) | 1.41(1.00-1.99) | 0.068 |  |
| **Current smoker** | |  |  |  |  |  |
| Yes | Ref | 1.24(0.88-1.77) | 0.98(0.66-1.45) | 1.00(0.64-1.56) | 0.514 | 0.195 |
| No | Ref | 0.94(0.73-1.23) | 0.95(0.72-1.26) | 1.09(0.80-1.50) | 0.412 |  |
| **Current drinker** | |  |  |  |  |  |
| Yes | Ref | 1.06(0.87-1.29) | 1.01(0.81-1.25) | 1.00(0.78-1.29) | 0.924 | 0.878 |
| No | Ref | 1.36(0.91-2.03) | 0.96(0.60-1.53) | 1.42(0.83-2.43) | 0.487 |  |
| **BMI** |  |  |  |  |  |  |
| <30 kg/m^2^ | Ref | 1.16(0.87-1.54) | 1.04(0.78-1.41) | 1.10(0.79-1.54) | 0.898 | 0.464 |
| ≥30 kg/m^2^ | Ref | 1.08(0.79-1.49) | 1.03(0.72-1.46) | 1.10(0.74-1.63) | 0.759 |  |

Adjusted for age, race/ethnicity, BMI, family income-poverty ratio, smoking status (not for smoke stratified analysis), drinking (not for drinking stratified analysis), leisure-time physical activity, total energy intake, hypertension, and diabetes.

Supplemental Table 2. Stratified analyses of the associations between dietary vitamin B12 intake with CVD in men

|  | **Quartile of dietary vitamin B12 intake** | | | | ***P_trend_*** | ***P_interaction_*** |
| --- | --- | --- | --- | --- | --- | --- |
|  | **Q1** | **Q2** | **Q3** | **Q4** |  |  |
| **Age** |  |  |  |  |  |  |
| <65y | Ref | 0.97(0.73-1.30) | 1.01(0.75-1.35) | 0.75(0.53-1.04) | 0.131 | 0.026 |
| ≥65y | Ref | 0.90(0.69-1.18) | 0.97(0.75-1.26) | 1.04(0.79-1.38) | 0.529 |  |
| **Current smoker** | |  |  |  |  |  |
| Yes | Ref | 0.73(0.53-1.02) | 0.81(0.58-1.12) | 0.80(0.56-1.15) | 0.372 | 0.102 |
| No | Ref | 1.01(0.80-1.29) | 1.05(0.83-1.33) | 1.01(0.78-1.31) | 0.898 |  |
| **Current drinker** | |  |  |  |  |  |
| Yes | Ref | 1.12(0.92-1.36) | 1.02(0.83-1.26) | 0.94(0.75-1.19) | 0.502 | 0.838 |
| No | Ref | 0.94(0.64-1.39) | 0.79(0.51-1.22) | 1.10(0.68-1.79) | 0.978 |  |
| **BMI** |  |  |  |  |  |  |
| <30 kg/m^2^ | Ref | 0.86(0.67-1.11) | 1.01(0.79-1.30) | 0.94(0.71-1.23) | 0.984 | 0.318 |
| ≥30 kg/m^2^ | Ref | 1.07(0.80-1.43) | 0.92(0.68-1.25) | 0.93(0.67-1.29) | 0.461 |  |

Adjusted for age, race/ethnicity, BMI, family income-poverty ratio, smoking status (not for smoke stratified analysis), drinking (not for drinking stratified analysis), leisure-time physical activity, total energy intake, hypertension, and diabetes.

Supplemental Table 3. Stratified analyses of the associations between dietary folate intake with CVD in women

|  | **Quartile of dietary folate intake** | | | | ***P_trend_*** | ***P_interaction_*** |
| --- | --- | --- | --- | --- | --- | --- |
|  | **Q1** | **Q2** | **Q3** | **Q4** |  |  |
| **Age** |  |  |  |  |  |  |
| <65y | Ref | 0.95(0.66-1.38) | 0.89(0.60-1.34) | 0.72(0.46-1.15) | 0.136 | 0.364 |
| ≥65y | Ref | 0.75(0.54-1.05) | 0.82(0.59-1.16) | 0.77(0.53-1.13) | 0.396 |  |
| **Current smoker** | |  |  |  |  |  |
| Yes | Ref | 0.85(0.61-1.18) | 1.05(0.74-1.49) | 0.86(0.57-1.29) | 0.833 | 0.251 |
| No | Ref | 0.84(0.58-1.21) | 0.74(0.51-1.09) | 0.57(0.37-0.88) | 0.007 |  |
| **Current drinker** | |  |  |  |  |  |
| Yes | Ref | 0.93(0.70-1.24) | 0.95(0.69-1.29) | 0.77(0.54-1.12) | 0.230 | 0.393 |
| No | Ref | 0.84(0.61-1.15) | 0.79(0.56-1.11) | 0.74(0.50-1.09) | 0.124 |  |
| **BMI** |  |  |  |  |  |  |
| <30 kg/m^2^ | Ref | 0.96(0.68-1.37) | 0.80(0.55-1.16) | 0.87(0.57-1.33) | 0.379 | 0.941 |
| ≥30 kg/m^2^ | Ref | 0.80(0.56-1.14) | 0.97(0.68-1.40) | 0.68(0.45-1.04) | 0.175 |  |

Adjusted for age, race/ethnicity, BMI, family income-poverty ratio, smoking status (not for smoke stratified analysis), drinking (not for drinking stratified analysis), leisure-time physical activity, total energy intake, hypertension, and diabetes.

Supplemental Table 4. Stratified analyses of the associations between dietary vitamin B12 intake with CVD in women

|  | **Quartile of dietary vitamin B12 intake** | | | | ***P_trend_*** | ***P_interaction_*** |
| --- | --- | --- | --- | --- | --- | --- |
|  | **Q1** | **Q2** | **Q3** | **Q4** |  |  |
| **Age** |  |  |  |  |  |  |
| <65y | Ref | 1.00(0.71-1.40) | 0.88(0.62-1.24) | 0.83(0.57-1.21) | 0.241 | 0.666 |
| ≥65y | Ref | 0.72(0.53-0.98) | 0.79(0.59-1.07) | 0.73(0.53-1.01) | 0.145 |  |
| **Current smoker** | |  |  |  |  |  |
| Yes | Ref | 1.00(0.73-1.37) | 1.09(0.80-1.50) | 1.08(0.78-1.51) | 0.536 | 0.098 |
| No | Ref | 0.72(0.52-0.98) | 0.61(0.44-0.84) | 0.54(0.38-0.77) | <0.001 |  |
| **Current drinker** | |  |  |  |  |  |
| Yes | Ref | 0.83(0.62-1.10) | 0.92(0.69-1.23) | 0.77(0.56-1.07) | 0.209 | 0.257 |
| No | Ref | 0.70(0.51-0.97) | 0.85(0.62-1.17) | 0.84(0.59-1.18) | 0.546 |  |
| **BMI** |  |  |  |  |  |  |
| <30 kg/m^2^ | Ref | 0.80(0.59-1.10) | 0.92(0.68-1.26) | 0.78(0.56-1.09) | 0.268 | 0.619 |
| ≥30 kg/m^2^ | Ref | 0.92(0.67-1.27) | 0.78(0.56-1.08) | 0.83(0.59-1.17) | 0.201 |  |

Adjusted for age, race/ethnicity, BMI, family income-poverty ratio, smoking status (not for smoke stratified analysis), drinking (not for drinking stratified analysis), leisure-time physical activity, total energy intake, hypertension, and diabetes.
